# A remarkable new species of the millipede genus *Trachyjulus* Peters, 1864 (Diplopoda, Spirostreptida, Cambalopsidae) from Thailand, based both on morphological and molecular evidence

**DOI:** 10.3897/zookeys.925.49953

**Published:** 2020-04-08

**Authors:** Natdanai Likhitrakarn, Sergei I. Golovatch, Ekgachai Jeratthitikul, Ruttapon Srisonchai, Chirasak Sutcharit, Somsak Panha

**Affiliations:** 1 Division of Plant Protection, Faculty of Agricultural Production, Maejo University, Chiang Mai 50290, Thailand Maejo University Chiang Mai Thailand; 2 Institute for Problems of Ecology and Evolution, Russian Academy of Sciences, Leninsky pr. 33, Moscow 119071, Russia Institute for Problems of Ecology and Evolution, Russian Academy of Sciences Moscow Russia; 3 Animal Systematics and Molecular Ecology Laboratory, Department of Biology, Faculty of Science, Mahidol University, Bangkok 10400, Thailand Mahidol University Bangkok Thailand; 4 Department of Biology, Faculty of Science, Khon Kaen University, Khon Kaen, 40002 Thailand Khon Kaen University Khon Kaen Thailand; 5 Animal Systematics Research Unit, Department of Biology, Faculty of Science, Chulalongkorn University, Bangkok 10330, Thailand Chulalongkorn University Bangkok Thailand

**Keywords:** cave, diplopod, molecular-based phylogeny, morphological character, taxonomy

## Abstract

A new, giant species of *Trachyjulus* from a cave in southern Thailand is described, illustrated, and compared to morphologically closely related taxa. This new species, *T. magnus***sp. nov.**, is much larger than all other congeners and looks especially similar to the grossly sympatric *T. unciger* Golovatch, Geoffroy, Mauriès & VandenSpiegel, 2012, which is widespread in southern Thailand. Phylogenetic trees, both rooted and unrooted, based on a concatenated dataset of the COI and 28S genes of nine species of Cambalopsidae (*Trachyjulus*, *Glyphiulus*, and *Plusioglyphiulus*), strongly support the monophyly of *Trachyjulus* and a clear-cut divergence between *T. magnus***sp. nov.** and *T. unciger* in revealing very high average *p*-distances of the COI gene (20.80–23.62%).

## Introduction

In South and Southeast Asia, as well as China, the juliform millipede family Cambalopsidae Cook, 1895 is among the most diverse, common, and often highly abundant groups that clearly dominate cave millipede faunas (Golovatch 2015). Four genera are actually involved.

By far the largest genus is *Glyphiulus* Gervais, 1847 with its 60+ species ranging across China and Southeast Asia to Borneo in the east (Golovatch et al. 2007a, 2007b, 2011b, 2011c, 2012b; Jiang et al. 2017, 2018; Likhitrakarn et al. 2017; Liu and Wynne 2019; Golovatch and Liu 2020). The genus *Plusioglyphiulus* Silvestri, 1923 encompasses 28 described species ranging from northern Thailand and Laos in the west, through Myanmar and Malaysia, to Borneo in the east and southeast (Golovatch et al. 2009, 2011a; Likhitrakarn et al. 2018). Interestingly, the famous Burmese amber, 99–100 Mya, appears to contain a typical *Plusioglyphiulus* yet to be described (Wesener in litt.). This is evidence both of the very old age of this genus and its long presence *in situ* (Likhitrakarn et al. 2018).

The genus *Hypocambala* Silvestri, 1895 is the smallest, but particularly widespread, presently containing 14 species in Southeast Asia, as well as scattered across several islands of the Pacific and Indian oceans (Golovatch et al. 2011d).

The more diverse genus *Trachyjulus* Peters, 1864 is currently known to comprise 32 described species (Golovatch et al. 2012a; Likhitrakarn et al. 2018). Most of them (80%) show restricted distributions and can be assigned to short-range endemics (geographic range ca 10,000 km^2^) (Harvey 2002). The genus ranges from Nepal, India, and Sri Lanka in the west, through Bangladesh and Myanmar, to Vietnam, Thailand, Peninsular Malaysia, Singapore, and Indonesia (Sumatra and Java) in the east (Golovatch et al. 2012a). Most *Trachyjulus* species have been recorded/described from a single locality/cave, but *T. ceylanicus* Peters, 1864, *T. dentatus* (Pocock, 1894), *T. singularis* (Attems, 1938), and *T. unciger* Golovatch, Geoffroy, Mauriès & VandenSpiegel, 2012 are relatively widespread, while *T. calvus* (Pocock, 1893) is nearly pantropical (Golovatch et al. 2012a).

During recent field surveys in southern Thailand, a new, unusually large *Trachyjulus* species was taken from a cave. From the first glance, it seemed to be particularly similar to the grossly sympatric *T. unciger*, but both are readily distinguished by body size and several other characters, including gonopodal structures. To better understand the species delimitations and their variations, we compare this new species to topotypes of *T. unciger* (Pung-Chang (= Tham Nam) Cave, Phang-Nga Province, Thailand) not only based on their morphological characters, but also on molecular evidence. In addition, molecular-based phylogenetic relationships within the genus *Trachyjulus* are revealed and discussed for the first time using mitochondrial cytochrome c oxidase subunit I (COI) and nuclear gene 28S rRNA sequences. These were obtained from para- or topotypes of nine species of Cambalopsidae, including not only five *Trachyjulus*, but also two *Glyphiulus* and two *Plusioglyphiulus* as outgroups. Two members of the family Harpagophoridae from the same order Spirostreptida, as well as two of the family Julidae, order Julida, are also included as more distant outgroups for tree rooting.

## Material and methods

### Sample collection

Specimens were collected from southern Myanmar and southern Thailand under the Animal Care and Use Protocol Review No. 1723018. The collecting sites were located by GPS by using a Garmin GPSMAP 60 CSx, and all coordinates and elevations were rechecked with Google Earth. Photographs of live animals were taken using a Nikon 700D digital camera with a Nikon AF-S VR 105 mm macro lens. The specimens collected were euthanized by a two-step method following AVMA Guidelines for the Euthanasia of Animals (AVMA 2013). Specimens were then preserved in 95% ethanol for morphological and molecular studies. Ethanol was replaced after 24 hours with fresh 95% ethanol to prevent their defensive chemicals from affecting future DNA extraction. Mostly para- or topotypes of six described species were also used for molecular analyses (Table 1).

The holotype, as well as most of the paratypes are housed in the Museum of Zoology, Chulalongkorn University (CUMZ), Bangkok, Thailand; a few paratypes have also been donated to the collections of the Zoological Museum, State University of Moscow, Russia (ZMUM) and the Natural History Museum of Denmark, University of Copenhagen, Denmark (NHMD), as indicated in the text.

### Morphological study

The specimens were examined, measured, and photographed under a Nikon SMZ 745T trinocular stereo microscope equipped with a Canon EOS 5DS R digital SLR camera. Scanning electron micrographs (SEM) were taken with a JEOL, JSM-5410 LV microscope using gold-coated samples, and the material returned to alcohol upon examination. Digital images obtained were processed and edited with Adobe Photoshop CS5. Line drawings were executed based on photographs and specimens examined under a Nikon SMZ 745T trinocular stereo microscope, equipped with a Canon EOS 5DS R digital SLR camera. The terminology used and the carinotaxic formulae in the descriptions follow those in Golovatch et al. (2007a, 2007b, 2012a), while body ring counts are after Enghoff et al. (1993) and Golovatch et al. (2007a).

### DNA extraction and molecular identification

Total genomic DNA was extracted from the dissected midbody ring tissues using the DNA extraction kit for animal tissue (NucleoSpin Tissue extraction kit, Macherey-Nagel, Germany), following the standard procedure of the manual. Fragments of the mitochondrial cytochrome *c* oxidase subunit I (COI, 690 bp) gene were amplified using LCO1490 (5'-GGTCAACAAATCATAAAGATATTGG-3'; Folmer et al. 1994) and hco outout (5'-GTAAATATATGRTGDGCTC; Schulmeister et al. 2002) or Nancy (5'-CCCGGTAAAATTAAAATATAAACTTC-3'; Bogdanowicz et al. 1993); while fragments of the nuclear 28S ribosomal RNA large subunit gene (28S) were amplified using primers 28F2-2 (5'-GCAGAACTGGCGCTGAGGGATGAAC-3') and 28SR2 GAGGCTGTKCACCTTGGAGACCTGCTGCG-3'; Passamaneck et al. 2004).

The PCR amplification was performed using a T100™ thermal cycler (BIO-RAD) with a final reaction volume of 20 μL (15 μL of EmeraldAmp GT PCR Master Mix, 1.5 μL of each primer, 10 ng of template DNA and distilled water up to 20 μL total volume). Thermal cycling was performed at 94 °C for 3 min, followed by 35 cycles of 94 °C for 30 s, annealing at 42–56 °C (depending on samples and the primer paired) for 60 s, extension at 72 °C for 90 s, and a final extension at 72 °C for 5 min. Amplification of PCR products were confirmed through 1.5% (w/v) agarose gel electrophoresis before purification by PEG precipitation. Purified PCR products were sequenced in both directions (forward and reverse) using an automated sequencer (ABI prism 3730XL). All nucleotide sequences in this study were deposited in the GenBank Nucleotide sequences database under submission numbers MN893771–MN893781 for COI, and MN897820–MN897826 for 28S. The collecting localities and submission codes of each nominal species are listed in Table 1.

### Phylogenetic analyses

Our phylogenetic analyses included a specimen (paratype) of *T. magnus* sp. nov. and six individuals of four previously described species, namely *T. singularis* (Attems, 1938), *T. phylloides* Golovatch, Geoffroy, Mauriès & VandenSpiegel, 2011, *T. bifidus* Likhitrakarn, Golovatch, Srisonchai, Brehier, Lin, Sutcharit & Panha, 2018, and *T. unciger* Golovatch, Geoffroy, Mauriès & VandenSpiegel, 2011. Specimens from other genera, i.e. *Glyphiulus sattaa* Golovatch, Geoffroy, Mauriès & VandenSpiegel, 2011, *G. duangdee* Golovatch, Geoffroy, Mauriès & VandenSpiegel, 2011, *Plusioglyphiulus erawan* Golovatch, Geoffroy, Mauriès & VandenSpiegel, 2011, and *P. saksit* Golovatch, Geoffroy, Mauriès & VandenSpiegel, 2011, were utilized as outgroups (Table 1). In addition, sequences of millipedes from distant diplopod families were retrieved from the GenBank database and included as more distant outgroups for tree rooting. These were both *Thyropygus bearti* Pimvichai et al., 2009 and *Thyropygus allevatus* (Karsch, 1881), representing the family Harpagophoridae, order Spirostreptida, as well as *Julus scandinavius* (Latzel, 1884) and *Brachyiulus pusillus* (Leach, 1815), both in the family Julidae, order Julida, as even more remote relatives.

**Table 1.** List of the species used for molecular phylogenetic analyses and their relevant information. * = paratype, ** = topotype.

Voucher number	Species	Locality	Geographical coordinates	GenBank accession numbers	
				COI	28S
CAM059*	*Trachyjulus bifidus* Likhitrakarn et al., 2018	Yae Gu Cave (River Cave), Tanintharyi, Myanmar	11°13'4.50"N, 99°10'32.33"E	MN893771	MN897820
CAM061*	*Trachyjulus bifidus* Likhitrakarn et al., 2018	Thin Bow Gu Cave (Linno Gu #2), Tanintharyi Region, Myanmar	11°11'23.0"N, 99°10'18.3"E	MN893772	MN897821
CAM027**	*Trachyjulus phylloides* Golovatch et al., 2012	Phra Kayang Cave, Ranong, Thailand	10°19'35.62"N, 98°45'53.54"E	MN893773	N/A
CAM079**	*Trachyjulus unciger* Golovatch et al., 2012	Pung-Chang Cave, Phang-Nga, Thailand	8°26'35.67"N, 98°30'57.32"E	MN893774	MN897822
CAM070*	*Trachyjulus magnus* sp. nov.	Wat Tham Khrom Wanaram, Surat Thani, Thailand	8°46'12.07"N, 99°22'6.36"E	MN893775	MN897823
CAM044*	*Trachyjulus singularis* (Attems, 1938)	Tham Kao Havot Cave, Chon Buri, Thailand	13°09'46.95"N, 101°35'51.97"E	MN893776	MN897824
CAM107**	*Trachyjulus singularis* (Attems, 1938)	Khao Loi Cave (Wat Ma Duea), Rayong, Thailand	13°03'27.00"N, 101°36'27.00"E	MN893777	MN897825
**Outgroup Cambalopsidae**					
CAM030*	*Glyphiulus sattaa* Golovatch et al., 2011	Tham Pum-Tham Pla Cave, Chiang Rai, Thailand	20°19'42.54"N, 99°51'50.12"E	MN893778	N/A
CAM022*	*Glyphiulus duangdee* Golovatch et al., 2011	Chan Cave, Uttaradit, Thailand	17°35'39.00"N, 100°25'18.30"E	MN893779	MN897826
CAM031*	*Plusioglyphiulus erawan* Golovatch et al., 2011	Erawan Cave, Lamphun, Thailand	18°19'37.79"N, 98°52'22.41"E	MN893780	N/A
CAM021*	*Plusioglyphiulus saksit* Golovatch et al., 2011	Tham Nennoi Cave, KhonKaen, Thailand	16°43'4.77"N, 101°53'39.08"E	MN893781	MN897826
**Outgroup Harpagophoridae (Sporostreptida)**					
CUMZ-D00057	*Thyropygus bearti* Pimvichai et al., 2009	Si-Chon, Nakhon Si Thammarat, Thailand	9°14'48.1"N 99°45'51.1"E	KC519519	N/A
CUMZ-D00021	*Thyropygus allevatus* (Karsch, 1881)	Siam-Nakorn-Thani village, Nakhon Si Thammarat, Thailand	8°25'23.9"N 99°58'07.0"E	KC519487	N/A
**Outgroup Julidae (Julida)**					
BIOUG22537	*Julus scandinavius* (Latzel, 1884)	Provincial Park, Ontario, Six Mile Lake, Canada:	44°53'52.8"N 79°45'25.2"W	MG320199	N/A
09BBMYR_083	*Brachyiulus pusillus* (Leach, 1815)	Gros Morne NP, Newfoundland and Labrador, Canada	49°25'37.2"N 57°44'20.4"W	KM611731	N/A

The sequences were edited and aligned using Clustal W, implemented in MEGA7 (Kumar et al. 2016). The aligned sequences were estimated for the best-fit model of nucleotide substitution for each gene separately by KAKUSAN4 (Tanabe 2011). Two phylogenetic methods, maximum likelihood (ML) and Bayesian Inference (BI), were implemented through the on-line CIPRES Science Gateway (Miller et al. 2010). The ML analysis was performed using RAxML v.8.2.10 (Stamatakis 2014) with 1,000 bootstrap replications and GTRGAMMA as the nucleotide substitution model (Silvestro and Michalak 2012). The BI analysis was performed by MrBayes 3.2.6 (Ronquist et al. 2012) using the Markov chain Monte Carlo technique (MCMC). The best-fit evolution models based on the Akaike Information Criterion (AIC: Akaike 1974) were applied: SYM+G for the 1^st^COI codon, and HKY85+G for the 2^nd^COI codon, the 3^rd^COI codon, and the 28S gene. Ten million generations were run with a random starting tree. The resultant trees were sampled every 1,000^th^ generation and were used to estimate the consensus tree topology; bipartition posterior probability (bpp) and branch lengths after the first 25% of obtained trees were discarded as burn-in. All Effective Sample Size (ESS) values sampled from the MCMC analysis were greater than 1,000 in all parameters. A neighbour-joining tree (NJ) based on K2P-distance was constructed based on the amino acid alignment of peptide sequences corresponding to the mitochondrial COI dataset. Interspecific genetic divergences based on the COI sequence were also evaluated using uncorrected *p*-distances. The NJ tree and *p*-distance were implemented in MEGA7 (Kumar et al. 2016).

## Taxonomic part

### Family Cambalopsidae Cook, 1895

**Genus *Trachyjulus* Peters, 1864**

#### Trachyjulus magnus sp. nov.

Taxon classificationAnimaliaSpirostreptidaCambalopsidae

110643D8-1E52-59E2-A981-E20FC4607843

http://zoobank.org/620CFB43-417C-4A18-A946-8DC27A5567B2

**Figure 1.**
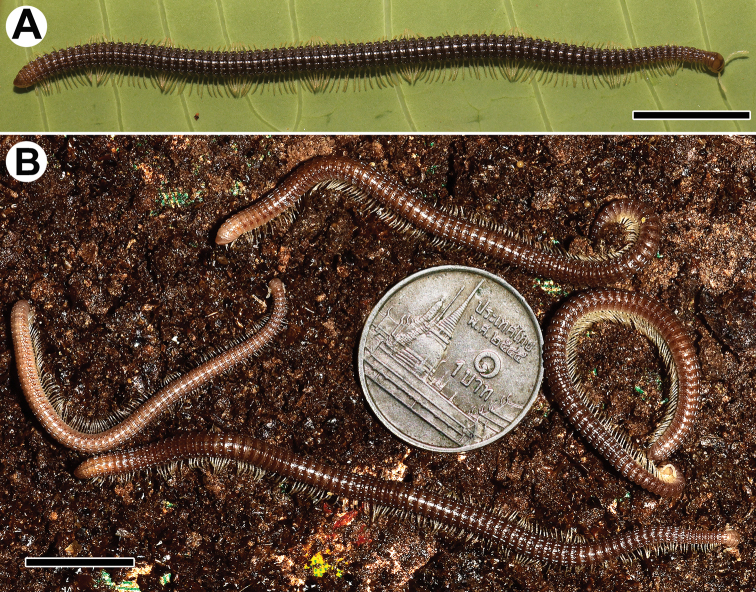
*Trachyjulus magnus* sp. nov., habitus, live coloration. **A** ♂ holotype **B** paratypes. Scale bars: 1 cm.

**Figure 2.**
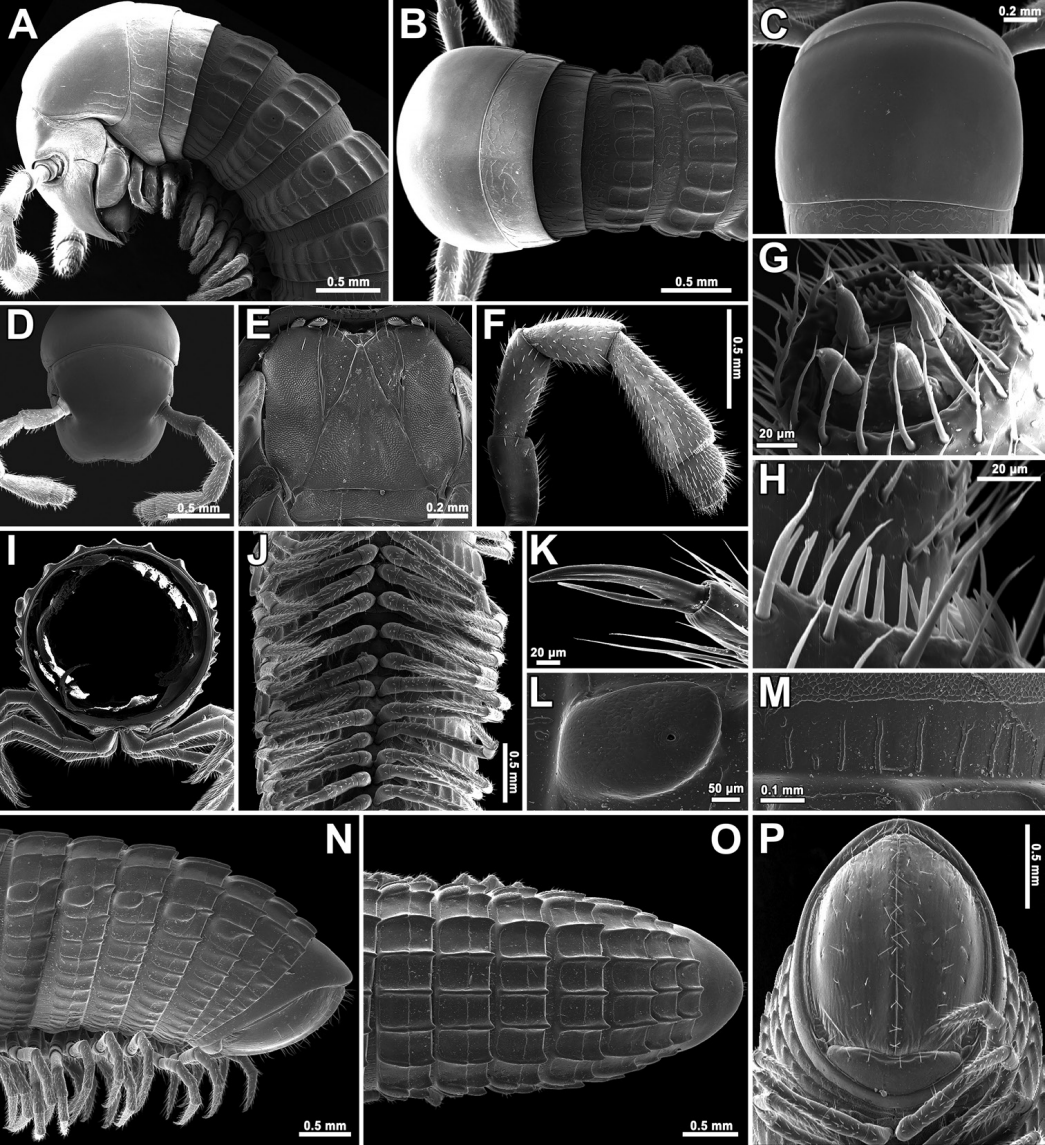
*Trachyjulus magnus* sp. nov., **A–C, I–P** ♀ paratype, **D–H** ♂ paratype. **A, B** anterior part of body, lateral and dorsal views, respectively **C** collum, dorsal view **D** cephalic capsule, dorsal view **E** gnathochilarium, ventral view **F** antenna, lateral view **G** tip of antenna **H** bacilliform sensilla on antennomere 5, lateral view **I** cross-section of midbody ring **J** midbody rings, ventral view **K** claw of midbody leg **L** enlarged ozopore region, lateral view **M** midbody prozona, dorsal view **N–P** posterior part of body, lateral, dorsal and ventral views, respectively.

**Figure 3.**
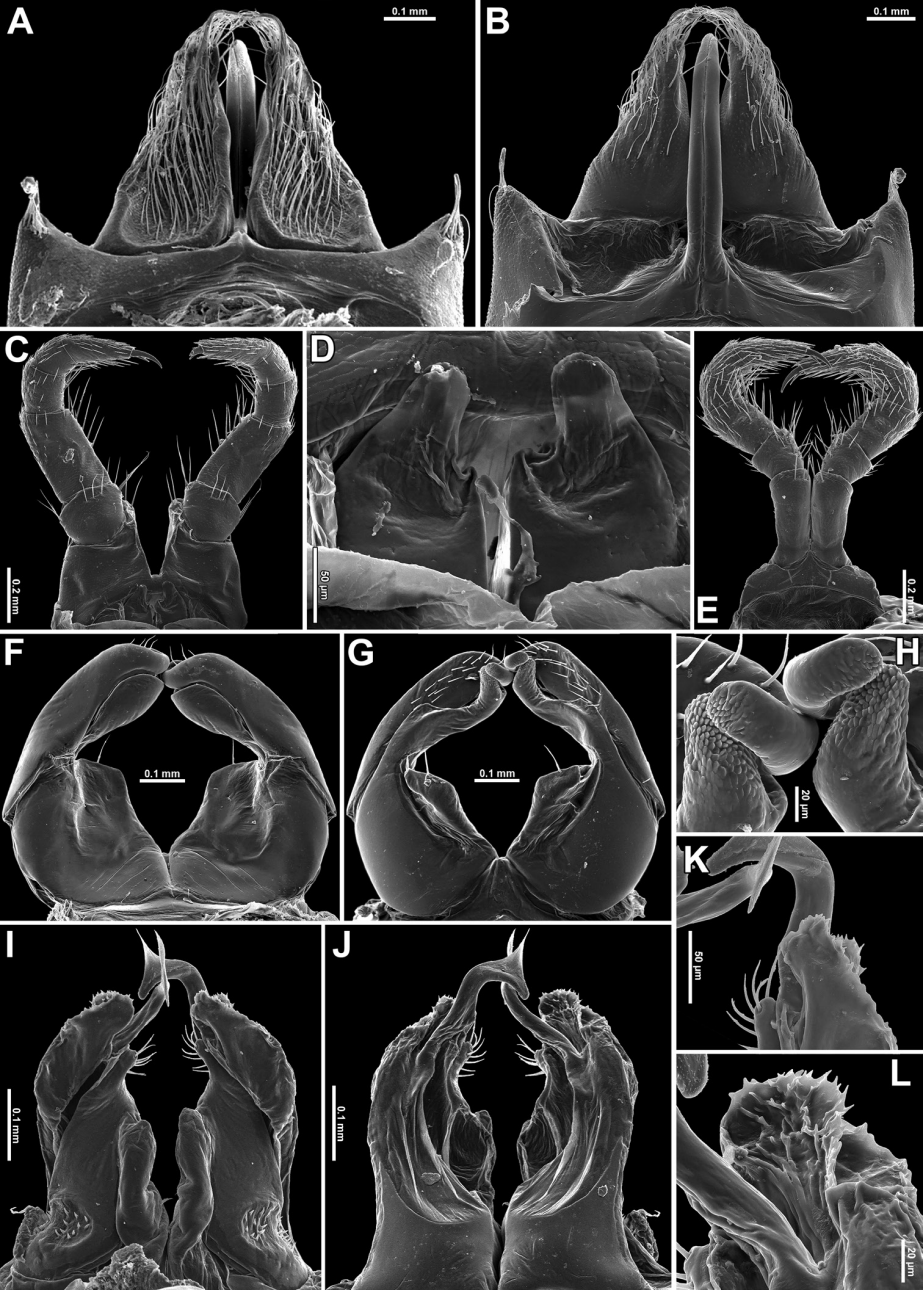
*Trachyjulus magnus* sp. nov., ♂ paratype. **A, B** Legs 1, frontal and caudal views, respectively **C** legs 2, caudal view **D** penes, caudal view **E** legs 3, frontal view **F, G** anterior gonopods, caudal and frontal views, respectively **H** telopodite tips of anterior gonopods **I, J** posterior gonopods, caudal and frontal views, respectively **K, L** telopodite tips of anterior gonopods, caudal and frontal views, respectively.

**Figure 4.**
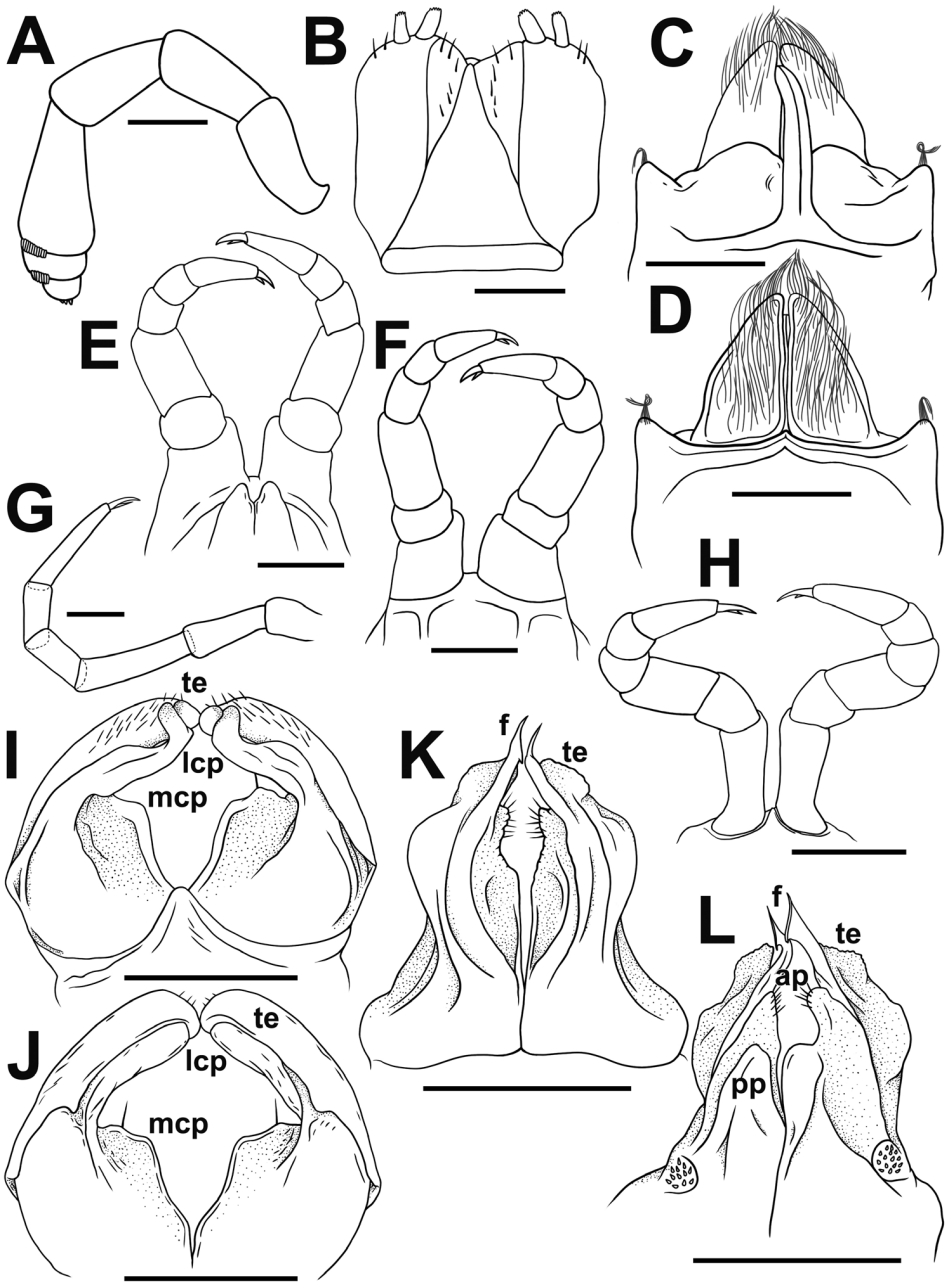
*Trachyjulus magnus* sp. nov., ♂ holotype. **A** Antenna, lateral view **B** gnathochilarium, ventral view **C, D** legs 1, caudal and frontal view, respectively **E, F** legs 2, caudal and frontal view, respectively **G** midbody leg, frontal view **H** legs 3, frontal view **I, J** anterior gonopods, frontal and caudal views, respectively **K, L** posterior gonopods, frontal and caudal views, respectively. Scale bars: 0.2 mm.

Figures 1 , 2 , 3 , 4

##### Type material.

***Holotype*** ♂ (CUMZ), Thailand, Surat Thani Province, Ban Na San District, Wat Tham Khrom Wanaram, 8°46'12.07"N, 99°22'6.36"E, 16.06.2018, leg. W. Siriwut, E. Jeratthitikul and N. Likhitrakarn.

***Paratypes.*** 15 ♂, 20 ♀ (CUMZ), 1 ♂, 1 ♀ (ZMUM), 1 ♂, 1 ♀ (NHMD), same locality, together with holotype.

##### Name.

To emphasize the largest body size of this species compared to all other species known in the genus.

##### Diagnosis.

This new species differs from all other *Trachyjulus* spp. by the largest body size (43.5–64.2 mm long, 2.1–2.8 mm wide), and also from the particularly similar and grossly sympatric *T. unciger* (23–42 mm long, 1.2–2.0 mm wide) in having the tegument of rings 2 and 3 nearly smooth (vs evidently carinate), carinotaxic formulae of typical rings (11–8/11–8+I/i+2/2+m/m vs 8–6/8–6+I/i+2/2+m/m), combined with the number of ommatidia (5–6+5–6 vs 4+4), and the posterior gonopods showing medial coxosternal processes (**mcp**) subtrapezoid (vs shorter and lobe-shaped).

##### Description.

Length of holotype ca 62.5 mm (Fig. 1A) and that of paratypes 44.1–64.3 (♂) or 43.5–64.2 mm (♀); midbody rings round in cross-section (Fig. 2I), their width (horizontal diameter) and height (vertical diameter) being similar; width of holotype 2.6 mm, of paratypes 2.1–2.7 (♂) or 2.1–2.8 mm (♀).

Coloration of live animals red-brown to yellow-brownish (Fig. 1), venter and legs brownish yellow to yellowish, antennae light to pale yellowish, eyes blackish, a thin axial line traceable; coloration in alcohol, after one year of preservation, similar, but body yellow-brownish to light brownish, vertex red-brown to light brown, eyes blackish to brownish.

**Figure 5.**
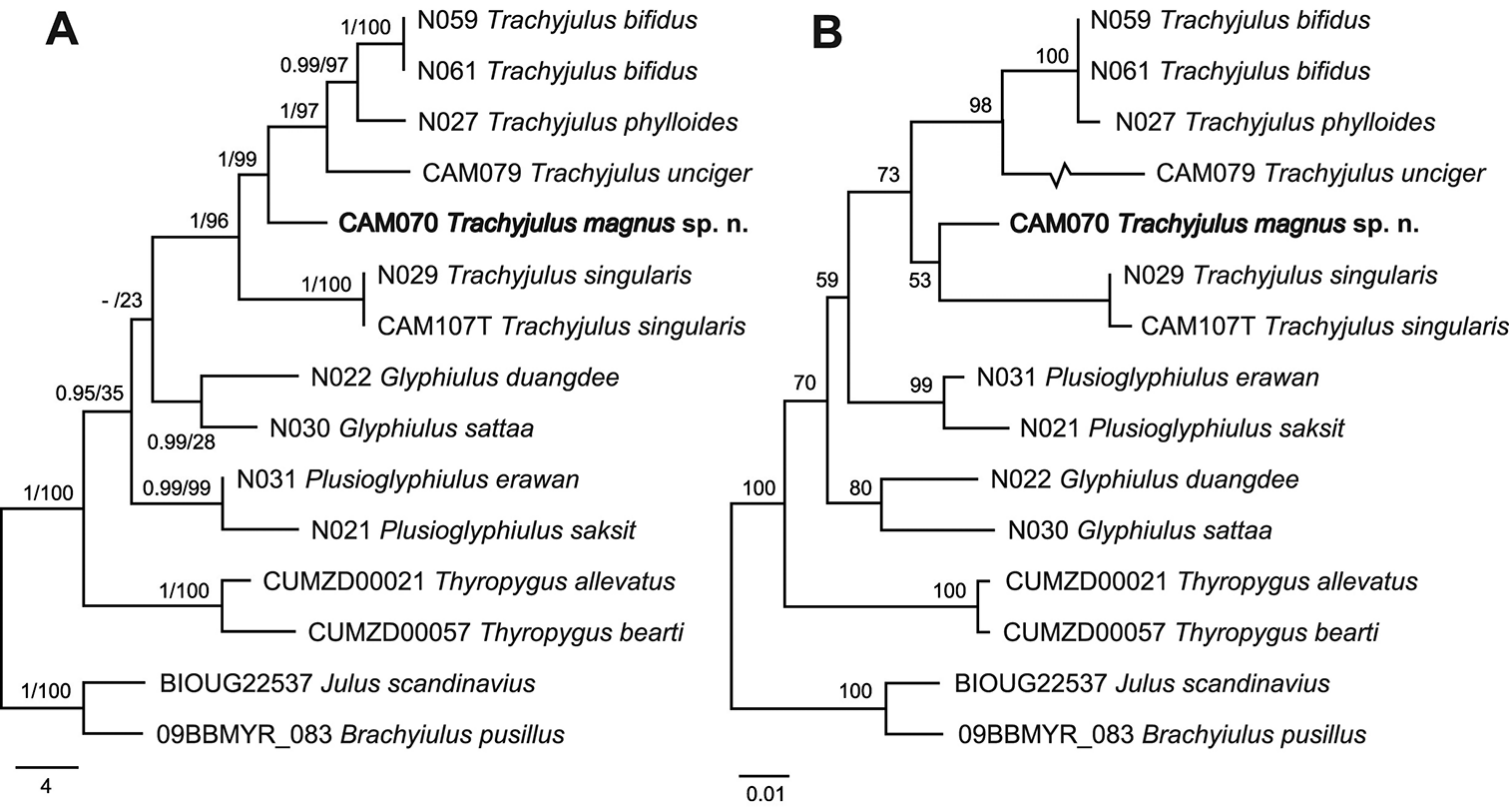
Phylogenetic analyses of *Trachyjulus* species and some related taxa. **A** Maximum likelihood tree based on a 1,501 bp alignment dataset of the nuclear 28S rRNA and mitochondrial COI genes. Numbers on nodes indicate bpp from Bayesian inference analysis (BI) and bootstrap values from maximum likelihood (ML), respectively **B** neighbour-joining tree (NJ) based on 230 amino acid alignments of peptide sequences corresponding to the mitochondrial COI dataset. Numbers on nodes indicate bootstrap values.

Body with 80p+2a+T rings (holotype); paratypes with 68–86p+1–3a+T (♂) or 69–93p+1–4a+T (♀) rings. Eyes large, flat, ovoid, with 6(5)+6(5) ommatidia arranged in a single vertical row (Fig. 2D). Antennae short and clavate (Figs 2A, B, D, F, 5A), extending past ring 4 laterally (♂, ♀), with four evident apical cones (Fig. 2G), antennomeres 5 and 6 each with a small distoventral group or corolla of bacilliform sensilla (Figs 2F, H, 5A). Clypeus with five teeth anteromedially (Fig. 2E). Gnathochilarium oligotrichous, mentum single (Figs 2E, 4B).

In width, head = ring 2 < ring 4 = 5 < 3 < 6 < 7 < 8 < 9 < 10 < collum = midbody ring (close to 12^th^ to 14^th^); body abruptly tapering towards telson on a few posteriormost rings (Fig. 2B, O). Postcollar constriction evident, but not particularly strong (Fig. 2B).

Collum (Fig. 2A–C) smooth, only near lateral edges with 2–4 light, short, superficial striae (Fig. 2A–C). Rings 2 and 3 nearly smooth, with 6–9 light, superficial striae (Fig. 2A, B). Following metaterga clearly and rather strongly carinate (Fig. 2A, B, N, O), especially so from ring 5 on, whence porosteles commence, these being completely absent from legless rings where ozopores are missing (Fig. 2A). Porosteles large, but low, conical, round, directed caudolaterad, broader than high (Fig. 2L). Carinotaxic formula of metaterga 4, 10–11/10–11+m/m (Fig. 2A, B). Carinotaxic formulae of following rings typically 11–8/11–8+I/i+2/2+m/m (Fig. 2A, B, N, O); all crests and tubercles, including porosteles, low. Tegument smooth (Figs 1, 2A, B, N, O), shining throughout. Fine longitudinal striations in front of stricture between pro- and metazonae, remaining surface of prozonae very delicately shagreened (Fig. 2A, B, M–O). Metatergal setae absent. Rings 2 and 3 each with long pleural flaps. Midbody ring nearly round in cross-section (Fig. 2I).

Epiproct (Fig. 2N–P) simple, bare, smooth, regularly rounded caudally. Paraprocts smooth, regularly convex and densely setose (Fig. 2P). Hypoproct as usual, transversely bean-shaped, slightly concave caudally (Fig. 2P).

Ventral flaps behind gonopod aperture on ♂ ring 7 barely distinguishable as low swellings, forming no marked transverse ridge.

Legs short, on midbody rings about 2/3 (♂, ♀) as long as body height (Figs 2I, J). Claw at base with a strong accessory spiniform claw almost half as long as main claw (Fig. 2K). Tarsi and tarsal setae very delicately fringed.

♂ legs 1 highly characteristic (Figs 3A, B, 4C, D), with a strongly enlarged, long, slim, central hook (actually a pair of very tightly adjacent) curved forward (Figs 3B, C, 4C), and strong, high, densely setose, triangular, 1-ringed telopodites (Figs 3A, B, 4E, F).

♂ legs 2 (Figs 3C, D, 4E, F) slightly enlarged, with high and large coxae; telopodites hirsute on anterior face; penes subconical, rounded apically, fused at base, bare.

♂ legs 3 (Figs 3E, 4H) slightly reduced, modified in having coxae especially slender and elongate.

Anterior gonopods rather simple (Figs 3F–H, 4I, J), with 1 or 2 strong apical setae on subtrapezoid, medial, coxosternal processes (**mcp**); telopodites (**te**) club-shaped, curved, sparsely setose, nearly as high as lateral coxosternal process (**lcp**), the latter slender and long, placed basal to telopodites. Anterior parts of lateral coxal processes and telopodites rod-shaped, slender and digitiform, with apicolaterally denticulate tips (Fig. 3H).

Posterior gonopods (Figs 3I–L, 4K, L) highly compact, coxites well separated from sternum, fused only basally, with a parabasal field of coniform microsetae caudally, each with a setose, paramedian, coxal process (**pp**) (Figs 3I, 4L); telopodites (**te**) high, distally microserrate/papillate (Fig. 3K, L); anterior coxal processes (**ap**) elongate, shorter than telopodites, densely setose and rounded distally (Figs 3I–K, 4K, L); both divided by a very high, axe-shaped flagellum (**f**) (Figs 3I, J, 4K, L).

##### Remark.

The often striking colour difference between head+collum+ring 2 and the remaining rings observed in SEM micrographs (Fig. 2) is certainly an artifact resulting from unwanted electrical charging.

## Phylogenetic analysis

Our concatenated dataset contained 15 individuals, including seven *Trachyjulus* ingroup and eight outgroup species, and an alignment of approximately 1,501 base pairs (bp). We were unable to obtain sequences of the 28S gene from *T. phylloides*, *G. sattaa*, and *P. erawan*. The final alignment of the COI gene fragment yielded 690 bp (298 variable sites, 270 parsimony informative), while the 28S gene fragment comprised 811 bp (100 variable sites, 45 parsimony informative). The phylogenetic tree estimated by both ML and BI revealed equivalent topologies. As only one position within the outgroup taxa was controversial, solely a ML tree is shown in Figure 5A. The monophyly of the genus *Trachyjulus* was strongly supported (1 bpp for BI and 96% bootstrap values for ML). Within the *Trachyjulus* clade, *T. singularis* was placed in the basal part, followed by *T. magnus* sp. nov., *T. unciger*, and a sister clade of *T. phylloides* and *T. bifidus*, respectively. All internal nodes were strongly supported (0.99–1 bpp for BI and 97–100% bootstrap values for ML). In addition, nine cambalopsid species were recovered as a monophyletic clade against the analyzed outgroups representing two other families and one order, although only the BI analysis was supported by this grouping and showed a bpp of 0.95. Within the cambalopsid clade, three genera were clustered separately as a monophyletic clade. However, no evolutionary relationship among them was revealed. The NJ tree based on the COI corresponding amino acid sequences also clearly recovered the monophyly of *Trachyjulus* (73% bootstrap values) (Fig. 5B).

The interspecific divergence of the COI uncorrected *p*-distance among these nine cambalopsid species was found to be generally high (13.48–24.49%; Table 2). Among the *Trachyjulus* species concerned, the average distance values ranged from 15.07–23.62%. *Trachyjulus singularis* showed the highest divergence from the other *Trachyjulus* species, ranging from 21.16–23.62%. The lowest divergence among *Trachyjulus* species was 15.07% between *T. bifidus* and *T. phylloides*. Five *Trachyjulus* species showed a long-distance relationship to their closely related genera, *Glyphiulus* (18.84–23.62%) and *Plusioglyphiulus* (20.00–24.49%). In addition, the average distances between the members of *Glyphiulus* and *Plusioglyphiulus* were also relatively high, ranging from 17.39–21.16%. The interspecific divergence among *Glyphiulus* and *Plusioglyphiulus* species amounted to 17.97 and 13.48, respectively.

**Table 2.** Matrix of the average interspecific genetic divergence (uncorrected *p*-distance: % ± SE) for the 690 bp barcoding region of the COI gene between *Trachyjulus* species and some related Cambalopsidae taxa.

Taxa	1.	2.	3.	4.	5.	6.	7.	8.
1. *Trachyjulus bifidus*								
2. *Trachyjulus phylloides*	15.07 ± 1.31							
3. *Trachyjulus unciger*	20.00 ± 1.51	19.13 ± 1.45						
4. *Trachyjulus magnus* sp. nov.	20.14 ± 1.52	20.00 ± 1.46	20.43 ± 1.52					
5. *Tachyjulus singularis*	21.16 ± 1.53	20.80 ± 1.50	23.62 ± 1.64	21.52 ± 1.54				
6. *Glyphiulus sattaa*	18.84 ± 1.50	17.68 ± 1.40	21.45 ± 1.58	18.12 ± 1.46	20.51 ± 1.53			
7. *Glyphiulus duangdee*	21.16 ± 1.51	21.16 ± 1.59	22.61 ± 1.56	23.62 ± 1.61	23.48 ± 1.59	17.97 ± 1.48		
8. *Plusioglyphiulus erawan*	21.74 ± 1.53	20.43 ± 1.46	24.49 ± 1.59	20.29 ± 1.48	21.45 ± 1.61	17.39 ± 1.40	19.42 ± 1.50	
9. *Plusioglyphiulus saksit*	20.72 ± 1.48	21.30 ± 1.44	24.06 ± 1.53	20.58 ± 1.51	20.00 ± 1.41	19.13 ± 1.43	21.16 ± 1.47	13.48 ± 1.26

## Discussion

*Trachyjulus magnus* sp. nov. clearly represents a taxonomically valid species based on both morphological and molecular evidence. In the latest taxonomic review of *Trachyjulus*, Golovatch et al. (2012a) emphasized and listed the following primary morphological characters deemed useful to distinguishing it from the other cambalopsid genera. The genus *Trachyjulus* shows a collum which is smooth or nearly smooth at least dorsally, usually not particularly inflated compared to postcollum constrictions; midbody metazonae are strongly carinate, the carinotaxic formulae typically being 11–8/11–8+I/i+2/2+m/m; male leg 1 is strongly reduced to a broad transverse coxosternum that shows a pair of central, often completely fused coxal processes flanked by rudimentary telopodites; some structures of the gonopods are also unique. It is gonopodal structures, often highly conservative, that usually appear to be especially useful for species delimitations among congeners in the family Cambalopsidae (Golovatch et al. 2007a, 2007b, 2009, 2011a, 2011b, 2011c, 2011d, 2012b; Jiang et al. 2017; Likhitrakarn et al. 2017).

Morphologically, the new species looks especially similar to *T. unciger*, but both are clearly distinguishable (see Diagnosis above). Molecular evidence likewise reveals a sufficiently strong genetic divergence between *T. magnus* sp. nov. and *T. unciger* (*p*-distance = 20.43±1.52) (Table 2). Compared to other studies, the interspecific distances among Bavarian millipedes range from >5% among members in the same genus and up to 33.18% between the different orders, averaging 14.17% (Spelda et al. 2011). In Thailand, Pimvichai et al. (2016) reported the interspecific divergences of mitochondrial COI as ranging between 2 and 17% in the large-bodied *Thyropygus* millipedes, family Harpagophoridae. The average interspecific divergences among *Trachyjulus* species in the present study appear to be even higher than in *Thyropygus*: 15.07–23.62%. This may be accounted for by the much smaller sizes of *Trachyjulus* spp., as well as their usually more limited dispersal capacities that make them largely restricted to a particular cave or cave complex (Golovatch et al. 2007a, 2011a). High rates of interspecific genetic differentiation in small-bodied cave-dwelling species have long been reported elsewhere: 8.2–9.2% between two parapatric Callipodida millipedes from the USA, *Tetracion tennesseensis* Causey, 1959 and *T. jonesi* Hoffman, 1956 (Loria et al. 2011).

The phylogenetic trees, both rooted and inrooted, and based on the concatenated dataset, provide strong support to the monophyly of the genus *Trachyjulus* in both ML and BI analyses (bpp = 1.0 for BI and bootstrap value = 100% for ML) (Fig. 5A). In addition, the NJ tree based on the COI corresponding amino acid sequences in which the protein evolves at a slow rate (Drummond et al. 2005) clearly recovered the monophyly of *Trachyjulus* as well (73% bootstrap values) (Fig. 5B). Therefore, the molecular evidence confirms that all of the *Trachyjulus* species concerned, including the new species, do belong to the same genus. Because the unrooted phylogram totally failed to alter the topology of the rooted one, only the latter is shown in Figure 5.

*Trachyjulus singularis* was recovered as the basal clade of the tree. It also showed the highest genetic divergence from the other *Trachyjulus* species (21.16–23.62%). These results are in accordance with their geographic distributions, as *T. singularis* occurs only in eastern Thailand, i.e. far away from the congeners in southern Thailand. In addition, *T. singularus* has retained the ancestral character of a divided promentum of the gnathochilarium, a trait absent from the other members of *Trachyjulus*, but present in two other related genera, *Glyphiulus* and *Plusioglyphiulus*.

In conclusion, we put on record the first results of a molecular phylogenetic study on *Trachyjulus*, a largely cavernicolous genus, using a combination of the nuclear 28S rRNA and mitochondrial CO1 genes for a total of 1,501 bp. Our results reveal high rates of interspecific divergence among *Trachyjulus* species and other closely related genera. Given that Thailand and the neighbouring countries are extremely rich in karst and karst caves, there can hardly be any doubt that additional new species of Cambalopsidae generally and *Trachyjulus* in particular still await discovery. A combination of morphological and molecular studies in Cambalopsidae seems the best to provide further insights into the taxonomy and phylogenetic relationships in this large and widespread group.

## Supplementary Material

XML Treatment for Trachyjulus magnus
